# Non-commercial pharmaceutical R&D: what do neglected diseases suggest about costs and efficiency?

**DOI:** 10.12688/f1000research.28281.1

**Published:** 2021-03-08

**Authors:** Marcela Vieira, Ryan Kimmitt, Suerie Moon

**Affiliations:** 1 Global Health Centre, Graduate Institute of International and Development Studies, Geneva, 1211, Switzerland

**Keywords:** research and development, product development, non-commercial R&D, commercial R&D, neglected diseases

## Abstract

**Background:** The past two decades have witnessed significant growth in non-commercial research and development (R&D) initiatives, particularly for neglected diseases, but there is limited understanding of the ways in which they compare with traditional commercial R&D. This study analyses costs, timeframes, and attrition rates of non-commercial R&D across multiple initiatives and how they compare to commercial R&D using the Portfolio-to-Impact (P2I) model as parameter of comparison.

**Methods:** This is a mixed-method, observational, descriptive and analytic study. We contacted 48 non-commercial R&D initiatives and received quantitative data from 8 organizations on 83 candidate products, and qualitative data through 14 interviews from 12 organizations.

**Results:** The quantitative data suggested that non-commercial R&D for new chemical entities is largely in line with P2I averages regarding total costs and timeframes, with variation by phase. The qualitative data identified more reasons why non-commercial R&D costs would be lower than commercial R&D, timeframes would be longer and attrition rates would be equivalent or higher, though the magnitude of effect is not known. The overall emerging hypothesis is that direct costs of non-commercial R&D are expected to be equivalent or somewhat lower than commercial, timeframes are expected to be equivalent or somewhat longer and attrition rates would be equivalent.

**Conclusions:** The study found that non-commercial R&D differs in many significant ways from commercial R&D. However, it is possible that the sum of these differences cancelled each other out such that total costs, timeframes and attrition rates were largely in line with P2I averages. Given the nascent area, with almost no prior literature focusing on costs, timeframes or attrition rates of non-commercial R&D initiatives, we see the merits of this study as generating hypotheses for further testing against a larger sample of quantitative data, and for understanding reasons underlying any significant differences between non-commercial and commercial initiatives.

## Introduction

The costs, timeframes and attrition rates of biomedical research and development (R&D) has long been of interest to scholars, practitioners and policymakers alike. These questions have recently gained increased salience in light of concerns about the potentially declining productivity of commercial R&D; missing technologies such as products for neglected diseases of poverty, antibiotics or outbreak-prone pathogens; and the high and rising prices of medicines such as those for cancers or rare diseases. Improved understanding of the biomedical R&D process is essential to address these societal challenges.

Relatedly, the question has arisen as to whether different approaches to organizing, financing or incentivizing R&D – sometimes referred to as “alternate” or “new” business models of R&D – can address some of the shortcomings of the traditional approach. One area where there has been significant experimentation in alternate business models is neglected diseases (ND) that predominantly affect people in low- and middle-income countries (LMICs). In addition to (usually early-stage) research taking place in academic or public institutes, later-stage product development for NDs has received increased funding and attention through the creation of about two dozen product development partnerships (PDPs) ^1^. With at least two decades of significant non-commercial R&D efforts behind us, primarily focused on the NDs, it is an opportune moment to examine more closely how they compare to traditional commercial R&D on costs and efficiency. Several studies of specific non-commercial R&D initiatives have been published ^2– 7^, but we did not find any research examining costs, timeframes or attrition rates across more than one initiative.

This study sought to contribute to the knowledge base by gathering evidence on the costs, timeframes and attrition rates of “non-commercial” R&D initiatives and analyse how they compared to estimates of commercial R&D. “Non-commercial” R&D is defined as *undertaken primarily with a not-for-profit purpose.* Often, the lead organizations of such initiatives are academic or governmental in nature, or non-profit PDPs. For-profit firms frequently play a collaborating role by providing access to compound libraries, technical expertise, and/or products for use in testing, among other in-kind contributions. However, these initiatives are not part of the firm’s core commercial portfolio or strategy, as they are not expected to generate significant (if any) market returns. Therefore “non-commercial” should not be interpreted as excluding the private sector. Furthermore, we prefer to use the broader term “non-commercial” rather than “non-profit” as in some cases a developer may earn profit or revenue on a product as a way to offset costs.

The Portfolio-to-Impact (P2I) tool was used as a parameter of comparison in the study. P2I is a modelling tool initially developed by TDR (Special Programme for Research and Training in Tropical Diseases) co-sponsored by the United Nations Children’s Fund (UNICEF), the United Nations Development Programme (UNDP), the World Bank and World Health Organization (WHO), to estimate funding needs to accelerate health product development from late stage preclinical study to phase III clinical trials, and to model potential product launches over time. A description of the tool is available elsewhere ^8^. This study was undertaken as part of a TDR-led consortium of organizations that conducted further analysis of the P2I model throughout 2019.

## Methods

The study was designed to collect: 1) quantitative data from non-commercial R&D initiatives on costs, timeframes and attrition rates, and 2) qualitative data from non-commercial R&D initiatives and/or experts on such initiatives to explain existing costs, timeframes and attrition rates and reasons why these might or might not differ from commercial R&D. Data was collected between June and September 2019. Participants were given the opportunity to review and comment on a first draft of the research report but were not allowed to withdraw their data (a copy of the consent form is available with the full research report as extended data; Annex 4, pp. 91–94 ^9^). All data has been aggregated and anonymized.

### Participant selection

The study population was selected using the database of pipeline technologies for neglected diseases developed by Duke University and Policy Cures Research ^10^. The database does not include all non-commercial R&D initiatives – for example, it excludes biodefense projects that are largely publicly-funded – but it is the most comprehensive database of which we are aware focusing on R&D for neglected diseases, which is by nature largely non-commercial. We used a version of the spreadsheet “Candidates in the pipeline for neglected diseases, as of August 31, 2017” sent to us by the authors, which included a categorization of the organizations by developer type. We selected all not-for-profit organizations that were directly involved in conducting R&D, which included product development partnerships (PDPs), academic and research institutions and public research institutes and other public sector organizations. There were a total of 443 candidate products and 285 organizations that fit this initial criterion. All 16 PDPs were included given their organizational focus on non-commercial R&D, and the list of PDPs was complemented by other studies ^1, 11^. In addition to PDPs, we included organizations that had at least one product that had reached Phase 3. Another 32 organizations were included after this second selection, and three additional organizations were included through snowball sampling. The final list of organizations is available in the full research report (extended data; Annex 1, pp. 77–78 ^9^). We contacted each organization by email, with the initial request addressed to the organization’s most senior executive (e.g. Chief Executive Officer, Executive Director, Managing Director) to ensure leadership was aware of and agreed to our interview request, as recommended by the ethical review process. In specific cases, where we had reason to know another employee would be relevant to or aware of our research project, we copied other individuals on the initial email. The senior executive often delegated the interview to one or more staff, such as the lead staff person responsible for R&D, finance, policy and/or external relations.

### Quantitative data – collection and analysis

We developed a questionnaire using MS Excel to collect quantitative data from the organizations in our sample on the costs, timeframes, and attrition rates for a given organization or project (extended data; Annex 2, pp. 79–89 ^9^). We also included quantitative data on costs, timeframes or attrition rates published in reports or articles prior to the start of this study from four organizations ^4– 7^. For timeframes, we consulted data available on the organizations’ websites and in the clinical trials database ClinicalTrials.gov.

We created a quantitative dataset in Excel combining data provided by respondents with publicly available information pertaining to the costs, timeframes, and attrition rates of non-commercial R&D initiatives. Data was anonymized and combined by product archetype. Due to the limitations of our dataset, we limited our analysis to only two P2I archetypes: simple and complex new chemical entities (NCE-Simple, NCE-Complex) ^12^. Only one organization provided information about diagnostics and two about vaccines (one only included aggregated totals for one product), and we excluded these from the analysis as it would be impossible to protect the anonymity of the organizations.

Several assumptions were made to standardize the data and allow comparison (see detailed methodology in extended data; pp. 31–32 ^9^). For costs, we assumed that money was spent at a steady rate across the time period of each phase. For data in currencies other than USD, a yearly exchange rate from the year in which the cost was incurred was used to make the conversion into USD. Totals were calculated in 2017 dollars to facilitate comparison with P2I model figures. Inflation and deflation adjustments used the standard consumer price index.

For timeframes, we calculated total time spent in development as the sum of time spent in Pre-clinical, Phase 1, 2 and 3. Phase 1a and 1b trials were counted as phase 1. Phase 2a, 2b, and 2c trials were counted as phase 2, as were phase 2/3 tests. Phase 3a and 3b are both counted as phase 3. An average of time per trial for each phase was taken to estimate the amount of time required for a given clinical phase. For some data points, early stage testing was aggregated for multiple drug candidates and total time spent in a phase was divided by the number of candidates. Many candidates have multiple trials in each phase and the average of all trials was used.

### Qualitative data – collection and analysis

A list of questions for semi-structured interviews was developed to collect the qualitative data (extended data; Annex 3, p. 90 ^9^). The questions were not pilot tested. Most interviews lasted about one hour and were either conducted in person in Geneva or using videoconferencing software and were recorded upon agreement of the participant. Interviews were held with individuals with a high degree of familiarity about product development from the organizations included in the study. Interviews were held by the three authors (MV- MPH, researcher, female; RK – researcher, male; SM – PhD., director of research, female) and no one else was present besides the participants and the researchers.

Transcription or notes from the interviews were analysed and coded using NVivo 12. Interviews were coded by MV based on themes derived from the data, and reviewed by SM. A description of the coding tree is not available. Interviews were anonymized both at individual and organization level and each was given a number (PO - participant organization) for quotation identification. Given the small sample size, data saturation was not reached.

### Parameter of comparison

We used parameters from the Portfolio-to-Impact (P2I) tool v2, which was initially developed by TDR ^8^ and updated by Duke University and Policy Cures Research ^10^. The P2I Model is based on assumptions of costs, timeframes and attrition rates. Assumptions on development costs at each phase were based on clinical trial costs from Parexel’s R&D cost sourcebook, derived from historical data on health product development of more than 25,000 candidates for all diseases, and further refined and validated by interviews. The underlying data used to construct those assumptions is not publicly available and it was not possible to disaggregate costs, timeframes or attrition rates by commercial vs non-commercial developer. Given that non-commercial R&D (in particular, non-commercial late-stage product development) is both relatively recent and small in scale, we assume that the vast majority of the data used to construct the P2I averages comes from commercial R&D. We compared our quantitative data to P2I averages, and our qualitative data compared non-commercial with commercial R&D.

We conducted a literature review on costs, timeframes and attrition rates of biomedical R&D to compare other estimates with those of the P2I Model (available at the Knowledge Portal on Innovation and Access to Medicines). There is a wide range of estimates, most focusing on the development of new chemical entities (NCEs) by pharmaceutical companies (commercial R&D). P2I averages are at the low end of the range of cost estimates available for commercial R&D ^13– 16^, are somewhat higher for phase 1, lower for phase 2 and within the same range for phase 3 timeframes ^14, 17– 19^ and success rates are lower for complex NCEs and in the same range for simple NCEs ^17, 18, 20– 23^. In comparison to the few estimates available for non-commercial R&D initiatives ^4, 24^, the P2I Model assumptions estimate lower success rates in all clinical development phases, while preclinical is higher. A more detailed literature review is available in the full research report.

## Results

We contacted a total of 48 organizations: 23 did not respond, 12 declined and 13 participated in some way (not all participating organizations provided both quantitative and qualitative data) - a participation rate of 27%. In total, we obtained quantitative data regarding 8 organizations and 83 products - 37 drug candidates (13 NCEs, 8 repurposed drugs and 16 not specified), as well as 19 vaccine and 27 diagnostic candidates. Qualitative data was obtained from 14 interviews with 20 individuals from 12 organizations; out of those, 18 individuals provided their perspective based on projects conducted within their own organizations and two were experts with knowledge of a range of organizations.

### Costs

**Figure 1.**
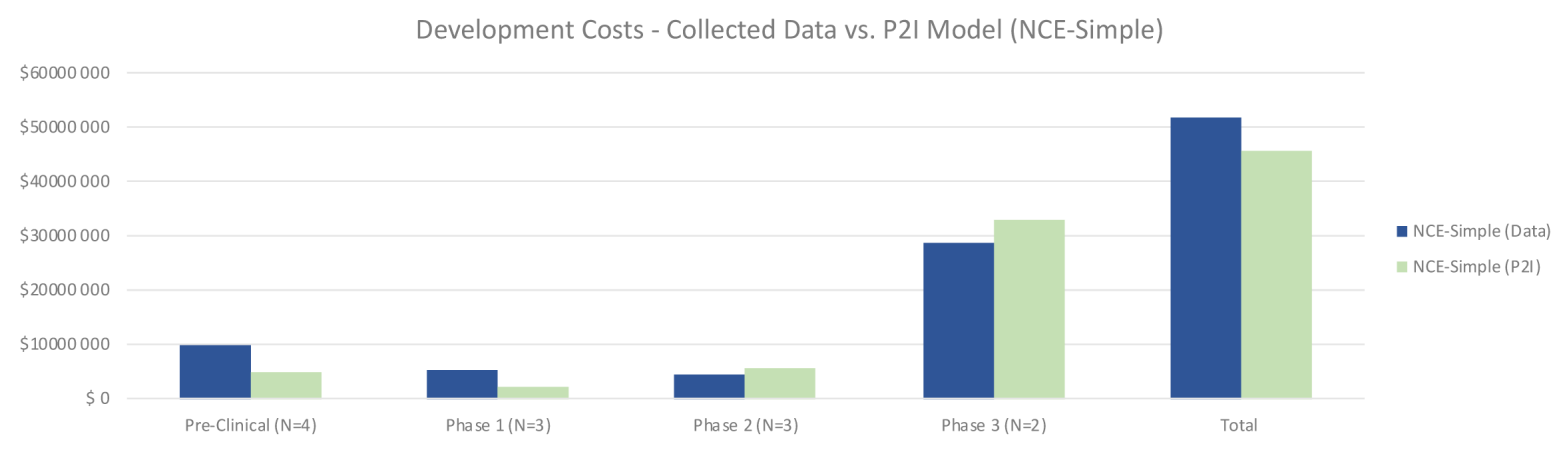
Development costs – collected data vs. P2I model (NCE-simple). The figure compares the development costs collected from the organizations in the study and the averages in the P2I Model for the archetype “new chemical entities – simple”. The comparison is made for different stages of development preclinical, phase 1, phase 2 and phase 3 and total costs. Data shows higher costs for collected data at preclinical, phase 1 and total, and lower costs at phase 2 and phase 3. Collected data for preclinical is based on 4 data points (n=4), for phase 1 n=3, for phase 2 n=3 and for phase 3 n=2.

Quantitative data on non-commercial R&D costs were largely in line with the P2I model estimates, with some variation by phase. For simple NCEs, total costs for non-commercial R&D were 13% higher than the P2I estimates (51.87 million USD for non-commercial vs 45.84 million USD for P2I) ( Figure 1). The largest differences were in pre-clinical and phase 1 – where the costs in our sample were more than double the P2I model estimates. Conversely, phase 2 and 3 trials were less expensive for simple NCEs in our data, but by a small margin. The sample size is too small for statistical significance or to generalize to non-commercial R&D more broadly; rather, the findings merely suggest a hypothesis that overall costs to develop simple NCEs are similar between non-commercial R&D initiatives and P2I averages.

**Figure 2.**
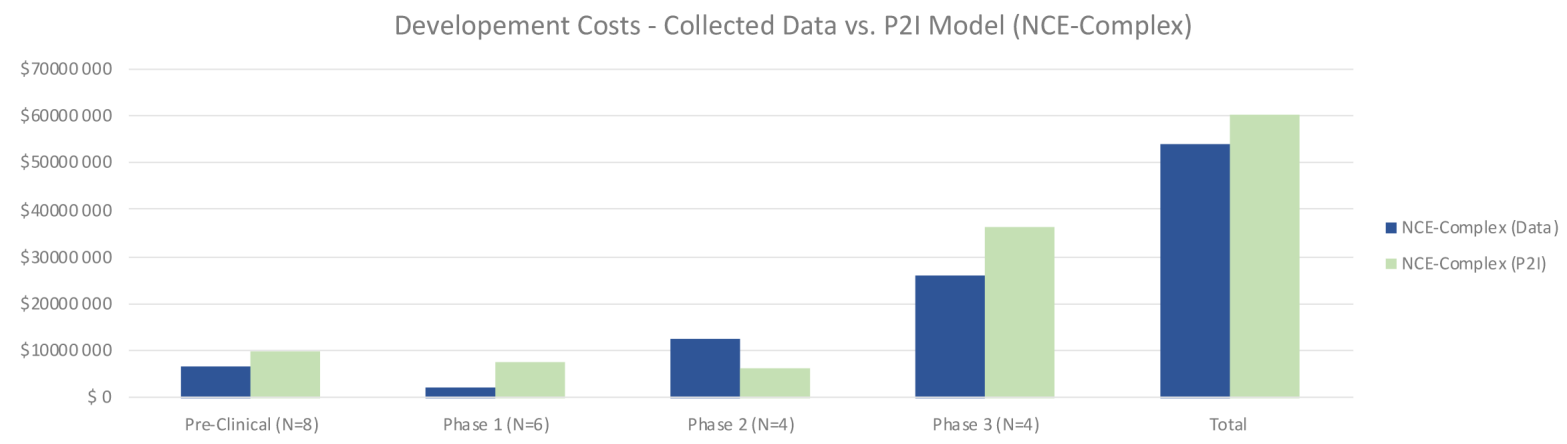
Development costs – collected data vs. P2I model (NCE-complex). The figure compares the development costs collected from the organizations in the study and the averages in the P2I Model for the archetype “new chemical entities – complex”. The comparison is made for different stages of development preclinical, phase 1, phase 2 and phase 3 and total costs. Data shows lower costs for collected data at preclinical, phase 1, phase 3 and total, and higher costs at phase 2. Collected data for preclinical is based on 8 data points (n=8), for phase 1 n=6, for phase 2 n=4 and for phase 3 n=4.

For complex NCEs, total costs were similar to the P2I model, 8% lower (53.98 million USD for non-commercial vs 58.93 million USD in P2I) ( Figure 2). In contrast with simple NCEs, for complex NCEs non-commercial preclinical and phase 1 costs were lower than the P2I model. Notably, phase 2 costs were much higher in our dataset (12.65 million USD vs 6.39 million USD in P2I). This could be in part because of the high proportion of phase 2/3 trials in the dataset, as well as the ratio of phase 2b to 2a tests being higher than the P2I data. Phase 3 costs were substantially lower than the P2I estimates, which may be explained by the fact that many pivotal trials were in phase 2. The opportunity to forgo phase 3 testing would drive up phase 2 costs while lowering phase 3 costs. The proportion of pivotal phase 2 tests may be different between P2I and our dataset. As with simple NCEs, the findings merely suggest a hypothesis that should be tested against a larger dataset – that overall costs to develop complex NCEs are similar between non-commercial R&D initiatives and P2I averages.

**Figure 3.**
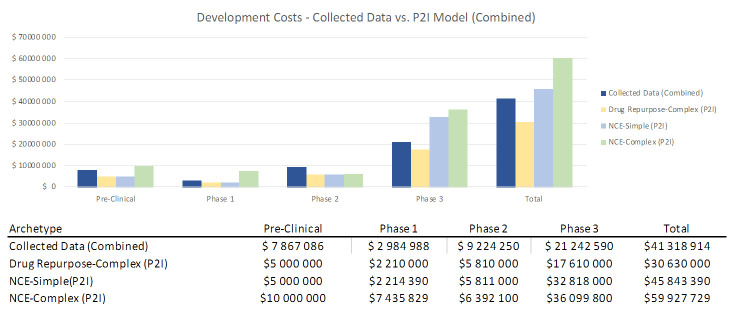
Development costs – collected data vs. P2I model (combined). The figure compares the development costs collected from the organizations in the study combining the archetypes “new chemical entities – simple” and “new chemical entities – complex” combined and the averages in the P2I Model for the archetypes “drug repurposed complex”, “new chemical entities – simple” and “new chemical entities – complex”. The comparison is made for different stages of development preclinical, phase 1, phase 2 and phase 3 and total costs.

To assess how sensitive our results were to coding by archetypes (i.e. characterizing a product as “simple” or “complex” NCEs), we combined our data from both categories and found that total costs (48.9m USD) lay between P2I’s NCE-simple and NCE-complex estimates (45.8m-59.9m USD) ( Figure 3). This sensitivity analysis suggests that total non-commercial costs are largely in line with P2I parameters, even if there are some differences in coding of archetypes.

The qualitative data identified 12 factors that drove costs up or down in the different phases of product development within non-commercial R&D initiatives ^9^ ( Table 1). Most responses focused on the clinical stages of development rather than pre-clinical or earlier. Three factors pushed costs upward, and five factors pushed costs downward for non-commercial R&D in comparison with commercial. Four factors were categorized as indeterminate as they would affect both non-commercial and commercial R&D in the same way. The table below presents a summary of the factors influencing costs. A description of the factors and sample quotes are available in the full research report.

**Table 1.** Factors influencing costs for non-commercial (vs commercial) R&D. The table lists the factors influencing costs for non-commercial research and development (R&D) in relation to commercial R&D. Factors are classified in three columns: “costs pushed upward”, “indeterminate” and “costs pushed downward”. There are three factors listed for pushing costs upwards, four factors as indeterminate and five factors pushing costs downward.

Costs pushed upward	Indeterminate	Costs pushed downward
Infrastructure building and training at LMIC trial sites	Number of arms of the trial	Type of technology (i.e. simpler)
Involvement of affected community in product development	Duration of treatment or disease progression	Trial location in LMIC (vs HIC)
Limited scientific understanding of the disease	Prevalence or incidence of the disease	Organisational costs (i.e. non-profits)
	Predictive model and attrition profile	Advance over standard of care easier to show with smaller trial size
		Lower input prices for non-profit organizations

There were more factors that would push costs for non-commercial R&D down vis-à-vis commercial models. However, as the qualitative data does not tell us about the magnitude of the effects, no firm conclusions can be drawn on whether non-commercial R&D would generally cost the same, less or more than commercial R&D.

### Timeframes

**Figure 4.**
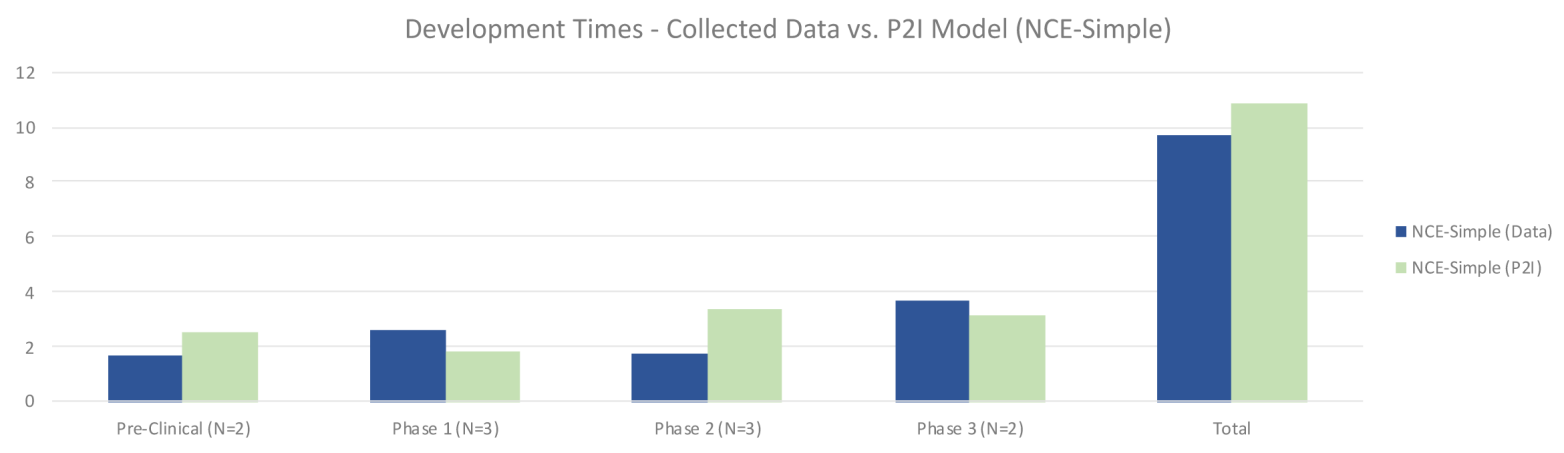
Development times – collected data vs. P2I model (NCE-simple). The figure compares the development times collected from the organizations in the study and the averages in the P2I Model for the archetype “new chemical entities – simple”. The comparison is made for different stages of development preclinical, phase 1, phase 2 and phase 3 and total costs. Data shows shorter times for collected data at preclinical, phase 2 and total, and longer times at phase 1 and phase 3. Collected data for preclinical is based on 2 data points (n=2), for phase 1 n=3, for phase 2 n=3 and for phase 3 n=2.

The quantitative data showed that for simple NCEs, timeframes between non-commercial R&D and P2I averages were roughly similar. Non-commercial R&D had shorter preclinical times (1.65 years vs 2.49 years in P2I), and longer phase 1 times (2.61 vs 1.80 years in P2I). Non-commercial R&D also had much shorter phase 2 times (1.75 vs 3.38 years in P2I), while phase 3 times were slightly higher (3.67 vs 3.18 years in P2I). Overall, our dataset suggested modestly faster timeframes for non-commercial simple NCE development, taking 9.67 years vs. 10.85 years in the P2I model ( Figure 4).

**Figure 5.**
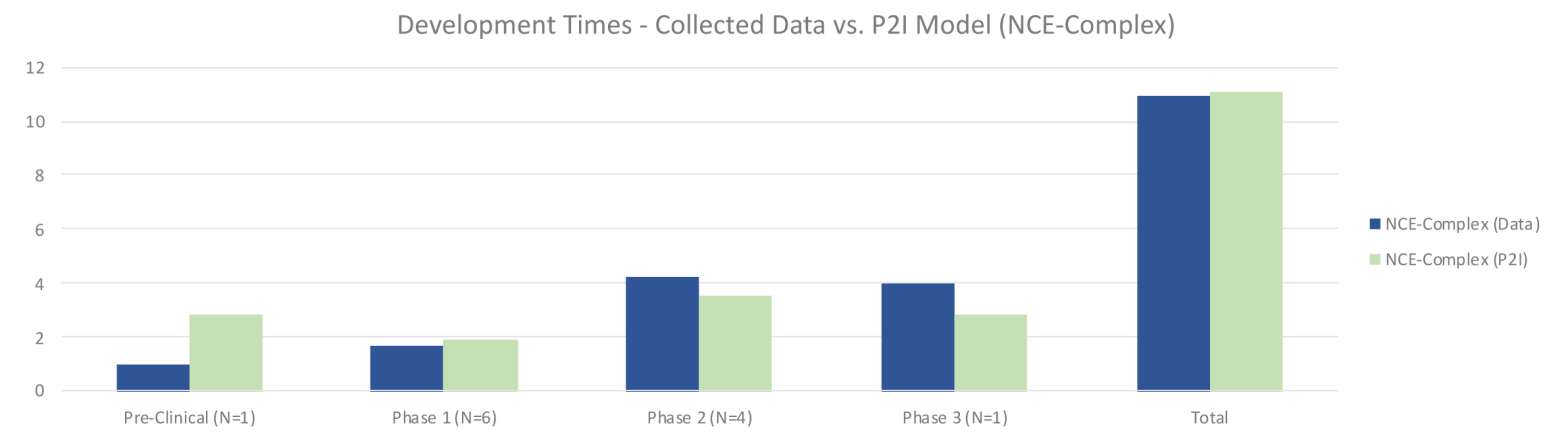
Development times – collected data vs. P2I model (NCE-complex). The figure compares the development times collected from the organizations in the study and the averages in the P2I Model for the archetype “new chemical entities – complex”. The comparison is made for different stages of development preclinical, phase 1, phase 2 and phase 3 and total costs. Data shows shorter times for collected data at preclinical, phase 1 and total, and longer times at phase 2 and phase 3. Collected data for preclinical is based on 1 data point (n=1), for phase 1 n=6, for phase 2 n=4 and for phase 3 n=1.

For complex NCEs, non-commercial pre-clinical testing was much shorter (1.00 vs 2.87 years in P2I), phase 1 testing slightly shorter (1.67 vs 1.93 years in P2I), phase 2 longer (4.25 vs 3.51 years in P2I), and phase 3 longer (4.0 vs 2.8 years in P2I) ( Figure 5). Overall, non-commercial development time was nearly identical, at 10.92 compared to 11.11 years for the P2I model. Possible explanations for these differences could be a result of the ratio of phase 2a to phase 2b tests included in phase 2. The P2I model does not suggest what proportion of its phase 2 tests are 2a compared to 2b, but for our data set, there were many phase 2b tests, which may have increased the amount of time in this phase. Pre-clinical time may have been shorter due to our decision to divide the time in this stage of development among multiple candidates, as that is how some data was shared with us. It is unclear how many preclinical studies in the P2I dataset would have been calculated in this way.

The qualitative data identified 12 factors influencing timeframes for non-commercial R&D ( Table 2). As with costs, the identified factors were categorized by their potential to push timeframes up or down for non-commercial R&D in comparison to commercial R&D. Seven factors were likely to lengthen timeframes for non-commercial R&D, no factors were likely to shorten timeframes and five factors were categorized as indeterminate. The table below presents a summary of the factors influencing timeframes. A longer description of the factors and sample quotes are available in the full research report ^9^.

**Table 2.** Factors influencing timeframes for non-commercial (vs commercial) R&D. The table lists the factors influencing timeframes for non-commercial research and development (R&D) in relation to commercial R&D. Factors are classified in three columns: “timeframes longer”, “indeterminate” and “timeframes shorter”. There are seven factors listed for longer timeframes, five factors as indeterminate and no factors for shorter timeframes.

Timeframes longer	Indeterminate	Timeframes shorter
Lower availability of funding	Need to develop regimens of multiple products (rather than single products)	
Slower decision-making processes	Combined Phase 2/3 trials	
Longer time to negotiate access to candidate compounds	Duration of treatment and/or disease progression	
Longer regulatory/ethical review	Seasonality of disease incidence	
Multiple simultaneous related trials, longer time to reach conclusions	Prevalence or incidence of the disease	
Smaller organizational scale or less mature organization		
Time for capacity building in LMICs		

There were a number of factors that would lengthen timeframes for non-commercial R&D vis-à-vis commercial models, or that were indeterminate ( Table 2). Notably, in none of the interviews did a respondent argue that non-commercial R&D would move faster. As the qualitative data does not tell us about the magnitude of the effect, no firm conclusions can be drawn on whether non-commercial R&D would take generally the same amount of time or more than commercial R&D.

### Attrition rates

The quantitative data on attrition rates was the most difficult to obtain, and there did not appear to be a standard methodology nor practice of calculating such rates within participating organizations. As all non-commercial initiatives in our sample had relatively small portfolios (compared to large commercial firms), attrition rates might not be meaningful. We judged that the data we received could not be aggregated across organizations, nor was it adequate for hypothesis generation. Further research is needed in this area.

Interviewees were also asked about the main factors that drive attrition rates up or down in the different phases of product development. This question generated a wide range of responses, and different organizations took quite different approaches to conceptualizing – let alone calculating – attrition rates. There was also reasonable disagreement as to whether or when a higher attrition rate is undesirable. Some interviewees argued that it is beneficial for an organization to “fail early and fail fast” – that is, to have a high(er) attrition rate in pre-clinical or Phase 1. Too low of an attrition rate could also suggest an organization is not taking enough risk, particularly in the earlier and lower-cost phases of R&D.

The qualitative data identified nine factors influencing attrition rates for non-commercial R&D ( Table 3). As with costs and timeframes, the identified factors were categorized as likely to drive attrition rates higher or lower for non-commercial R&D in comparison to commercial R&D. Three factors were identified as pushing attrition rates higher for non-commercial R&D, one factor as pushing attrition rates lower and five factors were categorized as indeterminate. The table below presents a summary of the factors influencing attrition rates. A longer description of the factors and sample quotes are available in the full research report ^9^.

**Table 3.** Factors influencing attrition rates for non-commercial (vs commercial) R&D. The table lists the factors influencing attrition rates for non-commercial research and development (R&D) in relation to commercial R&D. Factors are classified in three columns: “attrition rate higher”, “indeterminate” and “attrition rate lower”. There are three factors listed for higher attrition rates, five factors as indeterminate and one factor for lower attrition rates.

Attrition rate higher	Indeterminate	Attrition rate lower
Limited availability or use of optimization tools	Type of technology or product	Lower pre-existing standard of care means easier to demonstrate benefit of candidate product
Limited scientific understanding of disease	Testing for multiple indications	
Wide prevalence or incidence of the disease means broad target population across which a drug must be shown to be effective	Combinations or regimens	
	Reluctance to stop the project	
	Differing non-commercial vs commercial reasons for attrition	

There were more factors that would raise attrition rates for non-commercial R&D vis-à-vis commercial models, than would lower them, but most of the factors raised by respondents were indeterminate. As the qualitative data does not tell us about the magnitude of the effect, no firm conclusions can be drawn on whether non-commercial R&D would be characterized by higher, lower or equivalent attrition rates as commercial R&D.

## Discussion

The quantitative and qualitative data combined paint a complex, if grainy, picture. Keeping in mind the very small sample of quantitative data, the following hypotheses emerge from the analysis.

Regarding costs, the quantitative data suggest that non-commercial R&D total costs are about the *same* overall as P2I averages for NCEs. It should be noted that in comparison to other estimates available in the literature for commercial R&D, P2I averages are on the low end of the spectrum. The qualitative data identified many more reasons why non-commercial costs would be *lower* than commercial R&D, but did not shed light on the magnitude of the effects. The overall emerging hypothesis is that total direct costs of non-commercial R&D are expected to be *equivalent or somewhat lower* than commercial. Indirect costs for commercial R&D are expected to be higher due to higher overhead and capitalization costs.

Regarding timeframes, the quantitative data suggest that non-commercial R&D timeframes would be slightly *shorter* for simple NCEs and *equivalent* for complex NCEs in comparison to P2I averages. Yet the qualitative data identified many more reasons why non-commercial timeframes would be *longer* than commercial; the data did not shed light on the magnitude of the effects. The overall emerging hypothesis is that timeframes of non-commercial R&D are expected to be *equivalent or somewhat longer* than commercial.

Regarding attrition rates, the quantitative data was not adequate for analysis. The qualitative data uncovered more reasons why attrition rates might be higher in non-commercial R&D, but also provided a number of reasons why they might be lower or there might be no difference. Again, the magnitude of the effects is not quantified. The overall *very tentative* hypothesis that emerges is that attrition rates for non-commercial R&D would be *equivalent* to commercial R&D.

The study found that non-commercial R&D might differ in many significant ways from its commercial counterparts. However, it is possible that the sum of these differences cancels each other out such that total costs, timeframes and attrition rates would be largely equivalent to commercial R&D. If non-commercial R&D is characterized by equivalent or lower costs, equivalent or longer timeframes, and equivalent attrition rates to commercial R&D, the final expected direct costs and quantity of products resulting from a pipeline of non-commercially developed candidate technologies would be equivalent to those resulting from commercial R&D. In other words, the estimated parameters of the P2I v2.0 model are supported by this analysis, keeping in mind the differences between P2I averages and other estimates available in the literature for commercial R&D.

That said, this study identified a number of significant differences between non-commercial and commercial R&D. The many variables that affect cost, timeframes and attrition rates also highlight that caution is merited when comparing any single trial, product or organization against average benchmarks, as there are many legitimate reasons for departure from the mean. Therefore, the P2I model may need to be modified when applied more narrowly. While differences may get averaged out when the model is applied to a pipeline of nearly 450 candidates across a broad range of diseases (the intended use of the P2I model), they may be magnified in the narrower context of a single disease, technology type, or organization.

Finally, we re-emphasize that the small size and heterogeneity of the dataset means that these are *tentative* conclusions. Further quantitative research is needed to test these hypotheses against larger datasets. And further qualitative research is needed to deepen our understanding of the strengths and weaknesses of non-commercial R&D initiatives, and how well they function as alternatives to the traditional commercial model, especially beyond neglected diseases where commercial interests are higher.

## Conclusions

This was an observational, descriptive and analytic study of non-commercial R&D initiatives. The main limitations of the study were the small non-random sample size and the short period of time in which the study was conducted, which can partially explain the limited amount of quantitative data received. We also recognize that respondents may have had incentives to report costs, timeframes or attrition rates that were favourable to their organizations. Although we sought to check quantitative data against publicly available sources, in general very little relevant data was in the public domain or it was only available at a high level of aggregation. As a result, we have sought to be cautious in drawing inferences from the data.

Given the nascent nature of the area, with almost no prior literature focusing on costs, timeframes or attrition rates of non-commercial R&D initiatives, we see the merits of this study as generating hypotheses for further testing against a larger sample of quantitative data, and for providing intuition regarding reasons underlying any significant differences between non-commercial and commercial initiatives. The emerging hypothesis is that non-commercial R&D is comparable to commercial initiatives in efficiency, as indicated by direct costs, timeframes and attrition rates.

It is also important to highlight that many non-commercial R&D initiatives arose because the commercial model did not meet important global public health needs. This study did not compare the patient, population-level, equity or health system benefits offered by the products emerging from non-commercial vs commercial initiatives – only the costs, timeframes and attrition rates to develop those products. A fuller comparison could take both into account.

For future research, it may be useful both to expand the dataset on NCEs and also dedicate special attention to improving our understanding of non-commercial vaccine and diagnostics R&D, recalling that we excluded vaccines and diagnostics from our quantitative analysis due to very limited data, and were only able to examine a small sample for simple and complex NCEs.

Finally, in future research it would be useful to interview a broader range of stakeholders. Our interviews focused on practitioners with direct knowledge of non-commercial R&D initiatives involved in product development for neglected diseases, usually employees of the initiatives themselves. A more thorough picture is likely to emerge through interviews with additional non-commercial initiatives, and a broader range of their partners and funders.

## Data availability

The data referenced by this article are under copyright with the following copyright statement: Copyright: ï¿½ 2021 Vieira M et al.

Data associated with the article are available under the terms of the Creative Commons Attribution Licence, which permits unrestricted use, distribution, and reproduction in any medium, provided the original data is properly cited. 

### Underling quantitative data

Zenodo: Quantitative data: costs and timeframes_ non-commercial pharmaceutical R&D. https://doi.org/10.5281/zenodo.4519709 ^12^.

This project contains the following underlying data:

- Quantitative data_costs.csv
- Quantitative data_timeframes.csv
- README_quantitativedata_coststimeframes.txt

### Underlying qualitative data

The qualitative data is confidential to protect participant confidentiality as required by the Ethics Review Committee given the small number of organizations active in the field. Selected quotes from the interviews are available in the full research report at the Graduate Institute Institutional Repository at https://repository.graduateinstitute.ch/record/298834 ^9^.

### Extended data

The Graduate Institute Geneva Institutional Repository: Do costs, timeframes and attrition rates differ between non-commercial and commercial biomedical R&D ? A study of neglected diseases R&D and the P2I model. https://repository.graduateinstitute.ch/record/298834 ^9^. **

This report contains the following extended data:

- Consent form (Annex 4, pp. 91–94)
- The final list of organizations (Annex 1, pp. 77–78)
- Questionnaire for quantitative data (Annex 2, pp. 79–89)
- Assumptions made to standardize the data and allow comparison (pp. 31–32)

Data are available under the terms of the Creative Commons Attribution 4.0 International license (CC-BY 4.0).

## Authors contributions

Marcela Vieira - Conceptualization, Funding Acquisition, Methodology, Investigation, Data Collection, Data Curation, Formal Analysis, Writing – Original Draft Preparation

Ryan Kimmitt - Investigation, Data Collection, Data Curation, Visualization, Formal Analysis, Writing – Review & Editing

Suerie Moon - Conceptualization, Funding Acquisition, Methodology, Investigation, Data Collection, Formal Analysis, Supervision, Validation, Project Administration, Writing – Review & Editing
