# Supplementary material for: Non-commercial pharmaceutical R&D: what do neglected diseases suggest about costs and efficiency?
Source: F1000Res. 2021 Aug 16;10:190. Originally published 2021 Mar 8. [Version 2] doi: 10.12688/f1000research.28281.2 (PMC8063537; doi:10.12688/f1000research.28281.2)
Supplement: Peer review history

# Peer review: Reviewer response for version 2

**DOI:** 10.5256/f1000research.70804.r92005

**Published:** 2021-09-09

**Authors:** Lia Hasenclever

**Affiliations:** 1 Instituto de Economia, Universidade Federal do Rio de Janeiro, Rio de Janeiro, Brazil

I have read the answers presented by the author and consider them appropriate. They are enough for me to change my previous "Approved with Reservations" status.

Is the work clearly and accurately presented and does it cite the current literature?

Yes

If applicable, is the statistical analysis and its interpretation appropriate?

Not applicable

Are all the source data underlying the results available to ensure full reproducibility?

Yes

Is the study design appropriate and is the work technically sound?

Partly

Are the conclusions drawn adequately supported by the results?

Yes

Are sufficient details of methods and analysis provided to allow replication by others?

Yes

Reviewer Expertise:

NA

I confirm that I have read this submission and believe that I have an appropriate level of expertise to confirm that it is of an acceptable scientific standard.

# Peer review: Reviewer response for version 2

**DOI:** 10.5256/f1000research.70804.r92006

**Published:** 2021-08-17

**Authors:** Dzintars Gotham

**Affiliations:** 1 Medway NHS Foundation Trust, Gillingham, UK

Thank you for the detailed revisions and responses to my earlier review. I congratulate you on this very interesting study.

Is the work clearly and accurately presented and does it cite the current literature?

Yes

If applicable, is the statistical analysis and its interpretation appropriate?

Yes

Are all the source data underlying the results available to ensure full reproducibility?

Yes

Is the study design appropriate and is the work technically sound?

Yes

Are the conclusions drawn adequately supported by the results?

Partly

Are sufficient details of methods and analysis provided to allow replication by others?

Yes

Reviewer Expertise:

Pharmaceutical policy, health economics, global health

I confirm that I have read this submission and believe that I have an appropriate level of expertise to confirm that it is of an acceptable scientific standard.

# Peer review: Reviewer response for version 1

**DOI:** 10.5256/f1000research.31279.r82091

**Published:** 2021-04-19

**Authors:** Lia Hasenclever

**Affiliations:** 1 Instituto de Economia, Universidade Federal do Rio de Janeiro, Rio de Janeiro, Brazil

The article is of interest because there is a lot of controversy about R&D costs, and it brings new evidence. However, it does contain some inaccuracies.

 The most serious of these is the use of the term non-commercial. The use of this term is unusual, the most correct would be to use non-profit institutions. Furthermore, what the author calls offset costs, in fact, are indirect costs and not profits.

 I suggest using the conventional term It would be interesting to add a definition of what the author understands for each of the adopted comparison parameters: costs, timeframes and attrition rates (p….). This would make reading easier and reach a wider audience.

Is the work clearly and accurately presented and does it cite the current literature?

Yes

If applicable, is the statistical analysis and its interpretation appropriate?

Not applicable

Are all the source data underlying the results available to ensure full reproducibility?

Yes

Is the study design appropriate and is the work technically sound?

Partly

Are the conclusions drawn adequately supported by the results?

Yes

Are sufficient details of methods and analysis provided to allow replication by others?

Yes

Reviewer Expertise:

NA

I confirm that I have read this submission and believe that I have an appropriate level of expertise to confirm that it is of an acceptable scientific standard, however I have significant reservations, as outlined above.

# Response

**Published:** 2021-08-06

**Authors:** Marcela Vieira

**Affiliations:** Global Health Centre, Graduate Institute of International and Development Studies, Geneva, Switzerland

We thank you for your valuable comments and suggestions. We have amended the text to incorporate the changes and provide a point-by-point response to your comments below.

 1) The article is of interest because there is a lot of controversy about R&D costs, and it brings new evidence. However, it does contain some inaccuracies. The most serious of these is the use of the term non-commercial. The use of this term is unusual, the most correct would be to use non-profit institutions. Furthermore, what the author calls offset costs, in fact, are indirect costs and not profits.I suggest using the conventional term. 

 Response: We thank you for your suggestion. However, as explained in the introduction, we prefer to use the term non-commercial, instead of non-profit, as some organizations might earn profit, but that is not their main purpose for conducting product development. The term “profit” is used as the difference between the amount earned in revenues and the amount spent to bring a product to market. We removed the part on “offset costs” and rephrased the sentence for clarification. 

 2) It would be interesting to add a definition of what the author understands for each of the adopted comparison parameters: costs, timeframes and attrition rates (p….). This would make reading easier and reach a wider audience.

 Response: We added a definition of each of the terms.

# Peer review: Reviewer response for version 1

**DOI:** 10.5256/f1000research.31279.r82089

**Published:** 2021-04-06

**Authors:** Claudia Vaca-González

**Affiliations:** 1 Think Tank "Medicines, Information and Power", National University of Colombia, Bogotá, Colombia

This is a really valuable, well conducted and useful study. The report is well written and clear.

 Bellow some comments to improve the discussion and conclusions:

1. The study aims to generate hypotheses about the main differences between noncommercial and commercial initiatives. The overall emerging hypothesis is that timeframes of non-commercial R&D are expected to be equivalent or somewhat longer than commercial, but the authors do not comment any other factors that could impact this hypothesis, in order to conduct further studies, i.e.:
  - The size of the portfolio: a very reduced noncommercial portfolio that could become harder efforts to conduct the clinical studies in opposite with the commercial initiatives where the extended portfolio may leverage the efficiency.
  - The fulfillment of regulatory requirements: the commercial initiatives usually have an in-house or outsourcing regulatory and market access department to fulfill (and anticipate) any regulatory requirement. Is it the same in noncommercial initiatives? could these differences impact the total cost and the frame time of R&D?
2. There is not any critical appraisal of the used P2I model. Is it comprehensive at all? Is there any relevant factor that wasn't included or missed? 
  How much of the missed information is intrinsic related to the model complexity?
  Is it possible to become the model easier to fulfill?
  - I would not be worried about the size of the sample, more deeply qualitative data coming from experts and staffers could be rich on bring some other key factors to the table in order to understand the differences and similarities of noncommercial and commercial initiatives. Having that data, some researchers could rethink the models as P2I to make it easier to fulfill. I mean, to try to do the reverse investigation beginning at the end and go to the beginning.

Is the work clearly and accurately presented and does it cite the current literature?

Yes

If applicable, is the statistical analysis and its interpretation appropriate?

Not applicable

Are all the source data underlying the results available to ensure full reproducibility?

Yes

Is the study design appropriate and is the work technically sound?

Yes

Are the conclusions drawn adequately supported by the results?

Partly

Are sufficient details of methods and analysis provided to allow replication by others?

Yes

Reviewer Expertise:

Pharmacoepidemiology, regulatory issues and access-to- medicines public policy-making processes.

I confirm that I have read this submission and believe that I have an appropriate level of expertise to confirm that it is of an acceptable scientific standard.

# Response

**Published:** 2021-08-05

**Authors:** Marcela Vieira

**Affiliations:** Global Health Centre, Graduate Institute of International and Development Studies, Geneva, Switzerland

We thank you for your valuable comments and suggestions. We have amended the text to incorporate the changes and provide a point-by-point response to your comments below.

 1) The study aims to generate hypotheses about the main differences between noncommercial and commercial initiatives. The overall emerging hypothesis is that timeframes of non-commercial R&D are expected to be equivalent or somewhat longer than commercial, but the authors do not comment any other factors that could impact this hypothesis, in order to conduct further studies, i.e.:

 - The size of the portfolio: a very reduced noncommercial portfolio that could become harder efforts to conduct the clinical studies in opposite with the commercial initiatives where the extended portfolio may leverage the efficiency.

 - The fulfillment of regulatory requirements: the commercial initiatives usually have an in-house or outsourcing regulatory and market access department to fulfill (and anticipate) any regulatory requirement. Is it the same in noncommercial initiatives? could these differences impact the total cost and the frame time of R&D?

 Response: Thank you for your comments. The full research report (included as underlying data) provides more information about each of the factors mentioned in the interviews, including portfolio size, size of the organization and regulatory expertise. The full research report also includes selected quotes to better illustrate each point and how they might affect costs, timeframes and attrition rates. However, we chose to exclude these more detailed discussions from the article-length version of the study, in order to report the results in a more concise manner. 

 2) There is not any critical appraisal of the used P2I model. Is it comprehensive at all? Is there any relevant factor that wasn't included or missed?  How much of the missed information is intrinsic related to the model complexity? Is it possible to become the model easier to fulfill? I would not be worried about the size of the sample, more deeply qualitative data coming from experts and staffers could be rich on bring some other key factors to the table in order to understand the differences and similarities of noncommercial and commercial initiatives. Having that data, some researchers could rethink the models as P2I to make it easier to fulfill. I mean, to try to do the reverse investigation beginning at the end and go to the beginning.

 Response: Thank you for raising this discussion. The literature review and the quantitative and qualitative data collected under this study indicates that the underlying assumptions in the P2I Model for costs and timelines are in line with other estimates available in the literature and roughly similar to the data collected for non-commercial R&D. We believe that the qualitative data -- reported in more detail in the full research report -- does provide useful additional context regarding how the P2I model may be applied and interpreted, and would refer readers there. The data collected regarding attrition rates was not suitable for analysis and therefore the application of the full P2I model could not be validated. As discussed in the paper, the P2I Model may need to be modified when applied more narrowly to smaller and more specialized portfolios in the context of a single disease, technology type, or organization. Also, as mentioned in the introduction, our study was conducted as part of a TDR-led consortium of organizations that conducted further analysis of the P2I model throughout 2019. The other studies applied the P2I Model to their specific organizations and can be helpful for this discussion. That said, we believe that the Model is a useful tool and fairly easy to use as it requires only that the user inputs information about their average costs, timeframes and attrition rates (which can be modified according to the organization’s own figures). However, our study was not directly intended to suggest changes to the P2I Model and we believe that this discussion falls beyond the scope of the paper.

# Peer review: Reviewer response for version 1

**DOI:** 10.5256/f1000research.31279.r81104

**Published:** 2021-03-29

**Authors:** Dzintars Gotham

**Affiliations:** 1 Medway NHS Foundation Trust, Gillingham, UK

Dear Editor and authors,

 Thank you for the opportunity to review this interesting, well-designed, and important study. I suggest accepting with minor changes (treated in F1000Research as ‘approved with reservations'). My comments are mostly about interpretation/reporting rather than methodology.

 With kind regards,

 Dzintars

 ***

**Abstract:**

 Methods: not clear if it is 8+14 or 8 included in 14.

 Results: ‘more reasons’- more than what?  Oxford comma before “and attrition” could help readability.

 Conclusions: Suggest to start this with the headline finding: quantitatively costs and timeline were similar from survey and the P2I model. Currently drafting states attrition rates were also similar but this does not seem to be reflected in the text.

**Introduction:**

 First sentence should be, *“have long been”*.

 Some sources should be cited for the points in first paragraph regard declining productivity, e.g:

- Wieseler B, McGauran N, Kaiser T. New drugs: where did we go wrong and what can we do better? *BMJ* 2019; l4340.
- World Health Organization, World Intellectual Property Organization, World Trade Organization. Promoting Access to Medical Technologies and Innovation: Intersections between public health, intellectual property and trade. 2nd Edition. 2020 https://www.wto.org/english/res_e/booksp_e/who-wipo-wto_2020_e.pdf.

For the general reader, one or two more sentences explaining what NDs are would be useful, and that there has historically been next to zero private sector investment due to no prospect of return on investment. The same for the concept of PDPs.

 I would suggest the term ‘development time’ could be clearer than the term ‘timeframe’.

**Methods:**

 Para 1 – please delete ‘existing’ (what does this mean?).

 ‘simple’ and ‘complex’ NCEs are not standard concepts – please add a sentence explaining (or just a verbatim quote of how Terry/Yamey *et al. *defined them: Simple - Validated target or mechanism of action, Complex – Novel target or mechanism of action without understanding of disease pathogenesis). This could otherwise be misunderstood: in other contexts, there are ‘complex non-biologics’ (e.g. glatiramer), or, separately, the FDA concept of a ‘complex API’.

**Results:**

 In general: It would be helpful to have one more table, to summarize the quantitative findings all in one place (in addition to the useful bar charts that compare individual metrics).

 The qualitative data provide an interesting outline of the beliefs held by non-commercial drug developers regarding the comparison of their R&D process to commercial process. However, the comparison of ‘number of factors’ seems very tenuous. I would suggest to delete, *“There were more factors that would push costs for non-commercial R&D down vis-à-vis commercial models.”* as the authors acknowledge the number of ‘factors’ is meaningless in the following sentence. The following sentence ‘no firm conclusions can be drawn’ is also fairly self-evident and so would suggest the whole paragraph is deleted. The same for the analogous paragraphs in the following two sub-headings under the qualitative results (timeframes and attrition rates).

**Timeframes:**

 First sentence, please reword as *“…timeframes were roughly similar between…”*.

**Attrition rates:**

*“As all non-commercial initiatives in our sample had relatively small portfolios (compared to large commercial firms), attrition rates might not be meaningful. […] data we received could not be aggregated across organizations, nor was it adequate for hypothesis generation.”*

 Please provide a bit more details on your reasoning for not aggregating and not reporting the responses you received, and why you believe small portfolios would make rates not meaningful. Not intuitively clear to me.

**Discussion:**

*“The overall very tentative hypothesis that emerges is that attrition rates for non-commercial R&D would be equivalent to commercial R&D.”* I do not see any basis for this statement from the findings of the study, even with the ‘very tentative’ caveat.

 Development costs are hugely below what is reported by private industry – why? Also interesting to note that – my impression without looking up precise figures – development times are fairly similar to what industry reports, while costs are much lower. That makes intuitive sense as there is an inherent minimum development time needed for trials to see drug effects etc., while costs can be more labile and may be misreported.  

 Also, development costs seem substantially lower than DNDi report – why? (Drugs for Neglected Diseases initiative (DNDi) (2019), 15 Years of Needs-Driven Innovation for Access: Key Lessons, Challenges, and Opportunities for the Future, Geneva: DNDi.)

 You mention PDPs often develop products starting with a candidate compound ‘donated’ by private industry. Is it possible to sub-set which drugs came from a for-profit compound library and which didn’t?

 It would be worth touching upon, in the Discussion, why (whether?) it was necessary to keep respondent data anonymised and aggregated. Why do non-profits keep these data secret? Was it apparent to the authors, from preliminary discussions or from past experience, that non-profits would be unwilling to share data unless anonymised and aggregated? If so, interesting to the reader and valuable to future researchers to state this, I think.

 The quantitative part of the study effectively ends up being a validation exercise for the P2I model. That is valuable and interesting, but could be discussed a bit more in Background and Discussion – e.g. pointing out that P2I has not been previously externally validated (to my knowledge?) and the implications of the validation provided by this study, i.e. that P2I can indeed be used to plan ND R&D project funding.

Is the work clearly and accurately presented and does it cite the current literature?

Yes

If applicable, is the statistical analysis and its interpretation appropriate?

Yes

Are all the source data underlying the results available to ensure full reproducibility?

Yes

Is the study design appropriate and is the work technically sound?

Yes

Are the conclusions drawn adequately supported by the results?

Partly

Are sufficient details of methods and analysis provided to allow replication by others?

Yes

Reviewer Expertise:

Pharmaceutical policy, health economics, global health

I confirm that I have read this submission and believe that I have an appropriate level of expertise to confirm that it is of an acceptable scientific standard, however I have significant reservations, as outlined above.

# Response

**Published:** 2021-08-05

**Authors:** Marcela Vieira

**Affiliations:** Global Health Centre, Graduate Institute of International and Development Studies, Geneva, Switzerland

We thank you for your valuable comments and suggestions. We have amended the text to incorporate the changes and provide a point-by-point response to your comments below.

Abstract:

 1) Methods: not clear if it is 8+14 or 8 included in 14.

 Response: We thank you for pointing this out, and clarify that 8 is the number of organizations that submitted quantitative data, and 12 is the number of organizations that were interviewed for collection of the qualitative data (through 14 interviews). They might or might not be the same. For ease of comprehension, we decided to change the abstract to mention only the total number of organizations that participated in the study and left the detailed information in the “results” section.

 2) Results: ‘more reasons’- more than what?  Oxford comma before “and attrition” could help readability.

 Response: Thank you, we added a comma before “and attrition” for improved readability.

 3) Conclusions: Suggest to start this with the headline finding: quantitatively costs and timeline were similar from survey and the P2I model. Currently drafting states attrition rates were also similar but this does not seem to be reflected in the text.

 Response: Thank you for the suggestion, we changed the conclusions about attrition rate, and specified that the quantitative data suggested that costs and timelines are largely in line with commercial averages. 

Introduction:

 4) First sentence should be, “have long been”.

 Response: Thank you, we changed it accordingly.

 5) Some sources should be cited for the points in first paragraph regard declining productivity, e.g:

 Wieseler B, McGauran N, Kaiser T. New drugs: where did we go wrong and what can we do better? BMJ 2019; l4340.

 World Health Organization, World Intellectual Property Organization, World Trade Organization. Promoting Access to Medical Technologies and Innovation: Intersections between public health, intellectual property and trade. 2nd Edition. 2020 https://www.wto.org/english/res_e/booksp_e/who-wipo-wto_2020_e.pdf.

 Response: We thank you for the suggestions, however we believe that the first article focuses on another topic regarding the quality and benefits of new approved drugs, which is not a topic we discuss in our paper. We added the second reference in the introduction. 

 6) For the general reader, one or two more sentences explaining what NDs are would be useful, and that there has historically been next to zero private sector investment due to no prospect of return on investment. The same for the concept of PDPs.

 Response: We agree and added one sentence further explaining neglected diseases and another on PDPs.

 7) I would suggest the term ‘development time’ could be clearer than the term ‘timeframe’.

 Response: We thank you for the suggestion, and changed “timeframe” to “timeline” throughout the paper.

Methods:

 8) Para 1 – please delete ‘existing’ (what does this mean?).

 Response: Thank you for pointing this out, we deleted ‘existing’. 

 9) ‘simple’ and ‘complex’ NCEs are not standard concepts – please add a sentence explaining (or just a verbatim quote of how Terry/Yamey et al. defined them: Simple - Validated target or mechanism of action, Complex – Novel target or mechanism of action without understanding of disease pathogenesis). This could otherwise be misunderstood: in other contexts, there are ‘complex non-biologics’ (e.g. glatiramer), or, separately, the FDA concept of a ‘complex API’.

 Response: We thank you for the comment and have added the definitions as suggested. 

Results:

 10) In general: It would be helpful to have one more table, to summarize the quantitative findings all in one place (in addition to the useful bar charts that compare individual metrics).

 Response: Thank you for suggesting, we added a table summarizing the quantitative findings (new Table 5).

 11) The qualitative data provide an interesting outline of the beliefs held by non-commercial drug developers regarding the comparison of their R&D process to commercial process. However, the comparison of ‘number of factors’ seems very tenuous. I would suggest to delete, “There were more factors that would push costs for non-commercial R&D down vis-à-vis commercial models.” as the authors acknowledge the number of ‘factors’ is meaningless in the following sentence. The following sentence ‘no firm conclusions can be drawn’ is also fairly self-evident and so would suggest the whole paragraph is deleted. The same for the analogous paragraphs in the following two sub-headings under the qualitative results (timeframes and attrition rates).

 Response: We thank you for the valuable comment, however, as we are trying to develop intuition for hypotheses generation, we decided to keep the sentence stating the number of factors that were identified in one direction or the other, with the recognition that the magnitude of the effect of each factor is not known (as already stated). We believe that counting the number of factors that might differentiate commercial and non-commercial R&D is relevant for the discussion in the paper, and that we have accurately stated the limitations. We have, however, deleted ‘firm’ before conclusions, to state more forthrightly that no conclusions can be drawn.

 12) Timeframes:

 First sentence, please reword as “…timeframes were roughly similar between…”.

 Response: Reworded.

 13) Attrition rates:

 “As all non-commercial initiatives in our sample had relatively small portfolios (compared to large commercial firms), attrition rates might not be meaningful. […] data we received could not be aggregated across organizations, nor was it adequate for hypothesis generation.”

 Please provide a bit more details on your reasoning for not aggregating and not reporting the responses you received, and why you believe small portfolios would make rates not meaningful. Not intuitively clear to me.

 Response: We changed the text to better clarify why the data could not be aggregated (it was not reported by all organizations, and it was not calculated consistently across those that did)  and better explain the small portfolio effect (one or two failures lead to large swings in failure rates).

Discussion:

 14) “The overall very tentative hypothesis that emerges is that attrition rates for non-commercial R&D would be equivalent to commercial R&D.” I do not see any basis for this statement from the findings of the study, even with the ‘very tentative’ caveat.

 Response: We modified the discussion and conclusion regarding attrition rates to state that the data does not allow for hypothesis generation. 

 15) Development costs are hugely below what is reported by private industry – why? Also interesting to note that – my impression without looking up precise figures – development times are fairly similar to what industry reports, while costs are much lower. That makes intuitive sense as there is an inherent minimum development time needed for trials to see drug effects etc., while costs can be more labile and may be misreported.  

 Response: Thank you for your comment, we added more information about the parameter of comparison, further explaining that P2I averages are in line with other estimates for commercial R&D but there are a couple of estimates that are much higher (and added a table illustrating the range of estimates for costs - new Table 1). However, explaining the reasons why these estimates are so much higher is beyond the scope of the paper.

 16) Also, development costs seem substantially lower than DNDi report – why? (Drugs for Neglected Diseases initiative (DNDi) (2019), 15 Years of Needs-Driven Innovation for Access: Key Lessons, Challenges, and Opportunities for the Future, Geneva: DNDi.)

 Response: We added the DNDi report in the table illustrating the range of estimates for R&D costs (mentioned above). Explaining the reasons why the estimates differ among each other is beyond the scope of the paper. However, it is only preclinical costs in P2I that are lower than DNDi figures. DNDi costs for Phase I is in-between P2I NCE Simple and Complex and DNDi figures for Phase II and III combined are lower than P2I averages. We also flag in the conclusions the DNDi estimate for cost to bring one successful product to market, including cost of failures -- but this is a different figure from the per candidate/per phase numbers that P2I uses and that we report in the new Table 1.

 17) You mention PDPs often develop products starting with a candidate compound ‘donated’ by private industry. Is it possible to sub-set which drugs came from a for-profit compound library and which didn’t?

 Response: The quantitative data received does not allow for this disaggregation and the information about the origin of the compound was not provided.

 18) It would be worth touching upon, in the Discussion, why (whether?) it was necessary to keep respondent data anonymised and aggregated. Why do non-profits keep these data secret? Was it apparent to the authors, from preliminary discussions or from past experience, that non-profits would be unwilling to share data unless anonymised and aggregated? If so, interesting to the reader and valuable to future researchers to state this, I think.

 Response: As stated under “data availability”, the data was aggregated and anonymised to protect participant confidentiality as required by the Ethics Review Committee given the small number of organizations active in the field. Participant organizations were not asked if they would be willing to share their data openly, and we believe that this discussion falls beyond the scope of the paper.

 19) The quantitative part of the study effectively ends up being a validation exercise for the P2I model. That is valuable and interesting, but could be discussed a bit more in Background and Discussion – e.g. pointing out that P2I has not been previously externally validated (to my knowledge?) and the implications of the validation provided by this study, i.e. that P2I can indeed be used to plan ND R&D project funding.

 Response: Upon reflection, we have revised the paper to emphasize that we cannot draw any conclusions regarding attrition rates. Given the lack of data suitable for analysis regarding attrition rates, we have also amended our conclusions; our data does validate P2I estimates regarding costs and timelines, but does not validate the full P2I Model in terms of predictions for a portfolio of non-commercial R&D projects given the absence of attrition rate data. We added this in the discussion and conclusion sections. As we already discussed in the paper, the P2I model may need to be modified when applied more narrowly to smaller and more specialized portfolios in the context of a single disease, technology type, or organization. Also, as mentioned in the introduction, our study was conducted as part of a TDR-led consortium of organizations that conducted further analysis of the P2I model throughout 2019. The other studies applied the P2I Model to their specific organizations and can be helpful for this discussion.
